# Yellow Fever and Its Bacillus

**Published:** 1897-10

**Authors:** 


### EDITORIAL.

The office of the Business Manager of THE JOURNAL is 311 and 312 Fitten Building.

The editors are not responsible for views expressed by contributors.

Articles for publication should reach this office not later than the 15th inst.

Address all communications, and make all remittances payable to THE ATLANTA MEDICAL AND SURGICAL JOURNAL, Atlanta, Ga.

Reprints of articles will be furnished, when desired, at a small cost. Requests for the same should always be made *on the manuscript*.

### YELLOW FEVER AND ITS BACILLUS.

Some of the Gulf towns of Alabama, Mississippi and Louisiana are again invaded by yellow fever, the first case of the disease having been introduced into Ocean Springs from one of the West Indian Islands. From this point cases have radiated, until Biloxi, Scranton, Mobile, Edwards, Jackson and New Orleans have become infected, although the cases have been few in number and apparently mild in character. While it is not supposed that there is any danger, this late in the season, of a serious epidemic, the few cases that have appeared have been sufficient to demoralize business and to produce a wild exodus of the inhabitants from the infected districts. The occurrence of yellow fever so near home now gives additional interest to the reported discovery of the prime cause of the disease.

The observed facts concerning the spread of yellow fever furnished strong grounds for believing it of bacterial origin, but till now the organism has been sought in vain. Those familiar with bacterial work will readily appreciate the difficulties of the search. The chill, prostration, fever, vomiting and intestinal symptoms, the jaundice, hepatic degenerations and broken-down red corpuscles point to ptomainic poisoning. The partial immunity enjoyed by natives of the yellow fever belt seems to indicate the formation of an antitoxin and strengthens the idea of a bacterial origin.

It is well known that many varieties of bacteria are normally found in the intestinal canal. Many of these are necessary for well-being, but these innocuous species add to the difficulties of isolating a new form. It is not certain that the organism will be found in the intestinal canal despite the symptoms. Apomorphia acts as an emetic, but need not be in the stomach, and so a toxin elaborated anywhere may give intestinal symptoms. Again many diseases act in lower animals differently from the manner observed in man, and human experiments are rare. This makes it hard to prove our deductions.

The national importance of this disease has caused the federal government to give it attention, and Dr. Sternberg, our present Surgeon-General, was detailed to study its etiology and prevention. He studied the outbreaks in Havana in 1879, 1888 and 1889, Rio de Janeiro in 1887, Vera Cruz the same year, and Decatur, Ala., in 1888, and his report is the most complete monograph on the subject now in existence. It is chiefly negative, and disproves the claims of Carmona, Freire and others, to have isolated the specific organism. He suggested that a bacillus isolated by him in Havana in 1889, and called by him the bacillus X, might have some relation to the disease, but refrained from asserting it to be so until further evidence had been accumulated. In morphological and biological characters it resembles the bacillus coli communis, but differs widely from it in pathogenic powers. It does not liquefy gelatin, does not stain by Gram's method, and produces fermentation in glucose.

In an address before the University of Montevideo (Uruguay), in June of this year, Professor Sanarelli described a bacillus, called by him the bacillus icteroides, which he asserted to be the cause of yellow fever. The address is only available in the report of the *British Medical Journal* of July 3d, but from the account there given, it seems that it is identical with the bacillus X of Sternberg. As those who are not expert in bacteriology have difficulty in understanding technical descriptions, the points are shown in parallel columns:

| BACILLUS X. | BACILLUS ICTEROIDES. |
|---|---|
| 1. Isolated in "about half of cases." | 1. Isolated in 58 per cent. of cases. |
| 2. Small, oval, often in pairs. | 2. Short, generally united in pairs. |
| 3. Non-liquefying. | 3. Same. |
| 4. Facultative anaerobe. | 4. Same. |
| 5. Does not stain by Gram's method. | 5. Same. |
| 6. Growth abundant and of cream-like consistency. | 6. "Brilliant, opaque little drops like drops of milk." |
| 7. Pathogenic for rabbits and more so for guinea-pigs. | 7. Same. |

So far the evidence indicates identity, but the reports of pathogenic experiments differ. Many negative results followed Dr. Sternberg's inoculations; none followed those of Professor Sanarelli. But Dr. Sternberg says (*Amer. Jour. of Med. Sci.*, Sept., 1897), that he recorded a negative result when no symptoms appeared in five or six days, whereas Sanarelli says that his bacillus "in guinea-pigs, in very small or large doses, produces a cyclical febrile disease, which always ends in death after eight to twelve days," and this difference in time may explain the discrepancy. Sternberg (loc. cit.) frankly admits that had he obtained the results with his bacillus X that Sanarelli did with the bacillus icteroides, he would have asserted it to be the specific organism of yellow fever, and that if these results be confirmed by other observers the inference is unavoidable. The five human experiments seem conclusive. The cultures were made in bouillon, filtered through a Chamberlain bougie and sterilized with formalin. This made it really a solution of toxins, but in all five cases the symptoms of yellow fever followed.

Sanarelli's experiments also clear up an obscure point. It is well established that cold stops the disease, but as both the bacillus X and the bacillus icteroides survive freezing, it would appear that neither of them could cause the epidemic. Sanarelli showed that for the growth of the bacillus icteroides a preceding growth of a certain mould was necessary, just as the organism of lactic fermentation must precede that of butyric fermentation, and that this mould is sensitive to cold. Certain it is that we are nearer a solution of this grave problem, and that a great triumph is at hand for American pathology.

